# The effects of early childhood mental health consultation on early childhood teachers’ perceptions of children's challenging behaviors, expulsion risk, and the moderating role of teaching stress

**DOI:** 10.1002/imhj.70079

**Published:** 2026-03-23

**Authors:** Allison Boothe Trigg, Angela W. Keyes, Sarah Gray, Virginia Hatch, Kendyl T. Brunet, Sherryl Scott Heller

**Affiliations:** ^1^ Department of Psychiatry and Behavioral Sciences Tulane University School of Medicine New Orleans Louisiana USA; ^2^ Department of Psychological Sciences University of Connecticut, USA Storrs Connecticut USA; ^3^ Department of Psychology Tulane University New Orleans Louisiana USA; ^4^ Children's Bureau New Orleans Louisiana USA

**Keywords:** early care and education, infant and early childhood mental health consultation, teaching stress

## Abstract

The goal of this study was to explore the effects of infant and early childhood mental health consultation (IECMHC) on Louisiana early childhood teachers’ perceptions of individual children's and classroom behavioral challenges, children's resilience, and the risk of exclusionary practices (e.g., expulsion). It also examines how stress related to supporting a particular child (i.e., teaching stress) may influence IECMHC outcomes. A total of 194 early learning centers participated. Consultation services were provided in English at the program‐ and classroom‐level. Teachers reported expected positive shifts, including increased perceptions of child protective factors (e.g., self‐regulation) and reduced concerns related to individual and classroom behavior. Notably, reductions in classroom and child‐level behavioral concerns and teacher hopelessness and fear of accountability, as well as improvements in children's protective factors and self‐regulation, were most pronounced among teachers experiencing high levels of teaching stress. While findings are correlational, the role of teaching stress in shaping IECMHC outcomes offers insight into how these supports interact with expulsion risk. This information can guide the development of more effective, equity‐focused IECMHC programs that reduce exclusionary discipline practices and better support teacher well‐being and enhance the care of young children.

## 1 INTRODUCTION

In 2016 the US Department of Health and Human Services and the US Department of Education issued a policy statement highlighting the national data demonstrating that expulsions and suspensions were regularly occurring in preschool settings. This is problematic given the well‐established research indicating that expulsion and suspension contribute to several adverse outcomes across development, health, and education (American Psychological Association Zero Tolerance Task Force, 2008; Council on School Health et al., 2013; Skiba & Rausch, 2006; U.S. Department of Health and Human Services, 2016). Access to infant and early childhood mental health consultation (IECMHC) is related to lower rates of expulsion (Hepburn et al., 2013; Perry et al., 2011) and additional data are needed to document the specific pathways through which IECMHC reduces the risk of expulsion. The program evaluation data collected by a statewide IECMHC program examined the impact of a six‐month IECMHC intervention on expulsion and how teaching stress may moderate teachers’ perceptions of child behavior and subsequent expulsion risk.

### 1.1 Exclusionary discipline practices

Expulsion rates for children in preschool and prekindergarten programs differ significantly when compared to children in K‐12 settings. A seminal national study of nearly 4000 state‐funded prekindergarten classrooms found that four‐year‐olds were expelled at 3.2 times the rate of K‐12 students, with more than 10% of teachers reporting expelling at least one child and nearly 20% reporting expelling more than one child (Gilliam, 2005). Since that time, further research has demonstrated an alarming rise in the rates of expulsion across the nation, with estimates of roughly 250 instances of suspension or expulsion in both public and private preschools each day (Malik, 2017). Notably, boys and children of color are expelled at higher rates (Gilliam, 2005; National Center on Early Childhood Health and Wellness, 2022; US Department of Education, Office of Civil Rights, 2021). However, rather than leading to positive behavior change, these exclusionary practices are more often associated with negative outcomes in terms of behavior and academic achievement (Gerlinger et al., 2021).

When these concerning numbers were examined more closely, additional research uncovered a disturbing trend related to child demographics (e.g., age, gender, and race). Specifically, expulsion rates were about 50% higher for four‐year‐olds as compared to three‐year‐olds (Gilliam, 2005; US Department of Education Office of Civil Rights, 2016), and preschool‐aged boys are four times as likely to be expelled as preschool‐aged girls (Gilliam, 2005). Moreover, African American children are expelled almost twice as often as Latin American and white children and more than five times as often as Asian American children (National Center on Early Childhood Health and Wellness, 2022).

Taken together, these results suggest that the best predictors of preschool expulsion are the “three B's: big, Black and boy” (Zero to Three, 2017). While African American children comprise only 17.3% of preschool enrollment, they account for 30.6% of preschool suspensions and 24.6% of preschool expulsions. By comparison, non‐Latin American white preschoolers make up 43.4% of enrollment, but only 28% of suspensions (U.S. Department of Education Office of Civil Rights, 2021). These disparities are largely attributed to teachers’ implicit bias, which influences teachers’ perception of behavior without conscious awareness. Studies show that African American boys are often viewed as older than their actual age, less innocent, and more culpable than their same‐aged peers (Gilliam et al., 2016; Goff et al., 2014).

**Key Findings**

- After participating in infant and early childhood mental health consultation (IECMHC), teachers reported decreases in perceived child behavior difficulties, social emotional concerns, and expulsion risk along with increases in child protective factors.
- Teacher reporting high levels of pre‐consultation teaching stress (i.e., stress related to a specific child) reported significant decreases in perception of classroom level behavior difficulties after consultation, with their post‐consultation classroom behavior rating being similar to teachers with lower levels of pre‐consultation stress.
- Teaching stress (i.e., stress related to a specific child) may need to be a greater focus for programmatic IECMHC models to better support teachers and potentially impact systems that disproportionately utilize exclusionary methods of discipline and thereby reduce expulsion.

**STATEMENT OF RELEVANCE**

This study advances the field of infant and early childhood mental health by demonstrating how IECMHC may disrupt systems by reducing expulsion risk and teaching stress while enhancing teachers’ perception of child protective factors. By identifying teaching stress as a moderator of consultation outcomes, more tailored IECMHC approaches based upon teaching stress levels can be developed. IECMHC programs may use these findings to better identify the consultative support needs of teachers experiencing higher child‐specific stress in the classroom.

Subjecting children to exclusionary discipline practices has dire and profound short‐ and long‐term consequences. In the short‐term, children lose opportunities to learn and to interact with positive adult role models. They also miss out on chances to socialize with peers, which allows them to develop and practice social skills and emotion regulation. Children's self‐esteem can also be impacted, as they may view themselves as less intelligent, not capable of learning, and inherently bad, while also developing a negative impression about school and learning in general (American Psychological Association Zero Tolerance Task Force, 2008; National Center on Early Childhood Health and Wellness, 2022). When considering longer‐term consequences, research has shown that expulsion from school is associated with increases in later disciplinary action throughout the academic years, feeling disconnected from school, lower rates of graduation from high school (e.g., drop out or fail out), and greater rates of incarceration later in life (Adamu & Hogan, 2015).

Exclusionary discipline practices have wide‐reaching impacts for families. In recent studies, caregivers (e.g., biological parents, foster parents, grandparents) reported several financial impacts that resulted from exclusionary discipline, with some caregivers describing regularly receiving calls instructing them to pick up their children from their child care setting. As a result, caregivers missed work, often using sick and vacation time that was uncompensated; changed or reduced their work hours; lost work productivity; and sometimes had to quit or otherwise lost their jobs (Mitchell et al., 2016; O'Grady et al., 2024). While the child was suspended, caregivers were still responsible for paying the early childhood program while likely also paying for alternative child care, all of which led to a significant loss of income. Caregivers also described the toll on their emotional well‐being (e.g., feelings of anxiety, anger, fear, powerlessness, and rejection) as well as the impact on the family's support system that resulted from strained relationships with extended family and a loss of trust in the early childhood program. (O'Grady et al., 2024; Wahman et al., 2024).

Children whose teachers report them as having social emotional competence (e.g., demonstrate self‐regulatory behaviors, establish positive relationships, etc.) are less likely to be seen as difficult or challenging and thus less likely to be expelled. In addition, children's social‐emotional competencies are critical for school readiness and serve as protective factors that buffer children against stressors and future mental health issues (Bagdi & Vacca, 2005; Murano et al., 2020). IECMHC is one of the few evidenced‐based interventions that has been associated with improvements in children's social emotional competence (see Brennan et al., 2008; Perry et al., 2010; Silver et al., 2023; for reviews of IECMHC research literature).

### 1.2 Mental health consultation

One of the most promising strategies for reducing suspensions and expulsions in early childhood settings is having access to an infant and early childhood mental health consultant (MHC). Infant and early childhood mental health consultation strives to “build the capacity of staff, families, programs, and systems to prevent, identify, treat, and reduce the impact of mental health problems among children from birth to age six and their families” (Cohen & Kaufmann, 2000, p. 4). Mental health consultation can be viewed as a vital resource aimed at supporting early childhood providers in building their capacity to address challenging behaviors and promote healthy social‐emotional development (Heller et al., 2011). The relationships that children develop with their early childhood teachers is a critical component impacting the development of skills that are paramount to being ready to enter kindergarten. Positive child‐teacher relationships support children's creation of a positive self‐identity as they learn to successfully navigate social relationships and develop healthy skills to manage their emotions (Bowman et al., 2000). Children who are successful at developing social and emotional skills prior to entering school are more prepared for the demands of kindergarten (and beyond) and are more likely to be successful in kindergarten (Bowman et al., 2000; Shonkoff & Philips, 2000). A solid foundation of social and emotional literacy is associated with positive peer and adult relationships (LaFreniere & Sroufe, 1985) as well as fewer aggressive behaviors (Denham et al., 2002; Lemerise & Arsenio, 2000).

In a study by Gilliam (2005), teachers who received support from a mental health consultant demonstrated lower rates of expulsion. Subsequent research has further demonstrated the benefit of mental health consultation in decreasing expulsion rates. A 4‐year study designed to reduce the number of children expelled from centers in a large suburban county in Maryland found that 79% of children identified by their teachers as being at imminent risk for expulsion were able to remain in their current early childhood placement after their teachers worked with a mental health consultant (Perry et al., 2008). Another study on the effectiveness of IECMHC sought to isolate the effects of IECMHC for enhancing classroom quality and decreasing both teacher‐rated behavior problems and the likelihood of expulsion of teacher‐identified children (Gilliam et al., 2016). This randomized controlled trial compared classrooms and teachers assigned to receive IECMHC versus a waitlist control. Teachers in the IECMHC group identified the two children in their classrooms whose behaviors were most challenging to manage. After receiving three months of consultation, teacher ratings of externalizing behaviors decreased significantly. Overall, IECMHC is an approach that has gained greater recognition due to its ability to reduce exclusionary discipline practices, such as suspension and expulsion, in early childhood (Hepburn et al., 2013).

Though the evidence base supports that mental health consultation reduces exclusionary disciplinary practices, the mechanism of change by which this occurs is not clear. One avenue may be the impact that IECMHC has on teachers’ stress. Research has explored general stress that teachers, and in particular early childhood teachers, may experience. Predictors of teacher stress have been found at individual and environmental levels. Individual predictors include personal characteristics such as teacher age, race and gender, with teachers who are younger, male and BIPOC experiencing higher levels of stress (Friedman‐Krauss et al., 2014). Professional characteristics have also been found to contribute to higher levels of stress, including lower levels of education, fewer years of experience in the classroom, and low salary (Cumming, 2017). Teachers who report lower levels of teaching self‐efficacy (Jeon et al., 2018) and those who report higher levels of anger or anxiety (Ripski et al., 2011) demonstrate higher stress levels. Moreover, highly stressed teachers may be less equipped to maintain positive, responsive interactions with their students (Ansari et al., 2022), and highly‐stressed teacher profiles are associated with more disruptive student behaviors (Herman et al., 2017), which may contribute to the higher expulsion rates seen in early education settings. One study found that teachers who have requested expulsion within the past year more explicitly linked their stress to the behavior of an individual child, whereas teachers who had not recently requested expulsion reported sources of stress that were not specific to an individual child (Zinsser et al., 2019). Early care and education (ECE) teachers often experience stress due to limited benefits (e.g., low wages, limited or no paid time off and lack of professional respect), which may contribute to low levels of well‐being in the areas of physical health, mental health, and professional satisfaction (Corr et al., 2015; Cumming, 2017; Whitaker et al., 2013), while also bearing the responsibility for creating optimal caregiving and learning environments for our youngest and most vulnerable citizens. A study of working conditions among ECE teachers supports the notion that factors such as poor compensation and benefits packages, inadequate resources and support, suboptimal physical environments (e.g., lack of adult sized seating), and the overall demands required of them (e.g., lifting children, bending over to pick up toys, sweeping and mopping floors) contributes to high rates of teacher turnover and lower levels of classroom quality (Grant et al., 2019; Whitebook et al., 2014). It is important to consider the reciprocal nature of teacher stress in that teacher behavior simultaneously influences and is influenced by these factors both individually and multiplicatively. Thus, addressing teacher stress is of key importance for the early education field.

Gagnon et al. (2019) point to the difference between *teacher stress*, stress related to distal experiences as described above, and *teaching stress*, proximal stress that is related to a teacher's experience of working with a specific child. Teaching stress or stress related to an individual child's behavior may be related to teacher's perceptions of a child's behavior—adaptive or challenging—which may impact a child's risk of expulsion. Therefore, the path to reducing ECE expulsion rates and providing quality early care and education must take into consideration more than only teacher overall stress but also stress specific to certain children and how this teaching stress may be associated with a child's risk of expulsion.

### 1.3 The program

This statewide program of IECMHC to early education sites, TIKES, has been successfully implemented across Louisiana since 2007. To date, the TIKES IECMHC program has completed approximately 30,000 consultation visits to early education centers. The IECMHC program has three broad goals: (1) to support young children's social development, emotional development, and mental health; (2) to support adult caregivers’ abilities to support young children's healthy social development and emotional development; and (3) to design interventions and make appropriate referrals for children demonstrating developmental, behavioral, or mental health concerns. The program provides a combined model of IECMHC, meaning that child‐centered or classroom‐centered consultations that focus on individual children or classrooms are always conducted in the context of programmatic consultation that aims to improve the experience of all children, families, and staff connected to an early education site.

This IECMHC model consists of regular visits over a six‐month period to early education sites by a mental health professional with specialized training in infant and early childhood mental health as well as IECMHC. Sites receive two visits per month at every other week intervals. Consultants work with the site director to develop goals related to the consultation. The consultant provides MHC services to all teachers; however, the director leads the decision on which classrooms will be a focus. The consultants spend time on the following primary activities: observing and modeling in the classroom, meeting with teachers, meeting with parents, providing professional development opportunities, conducting child‐centered consultations, and collaborating with other support personnel. After the completion of a six‐month period of IECMHC, a site with seven or fewer classrooms will have received approximately 12 visits and a site with eight or more classrooms will have received approximately 24 visits. Each visit lasts between three and six hours depending upon center size and need.

All consultants are licensed or license‐eligible mental health professionals. License‐eligible consultants must be working under clinical supervision. Most consultants are licensed clinical social workers or licensed professional counselors. Each consultant receives twice monthly individual and once monthly group reflective supervision from a senior mental health consultant within the program with expertise in IECMHC as well as experience and training providing supervision (both clinical and reflective). The consultation program is funded through the state Department of Education, which contracts with a private university to administer IECMHC services to eligible early education programs across the state. Each consultant is hired through a subcontracted regional social service oriented non‐profit organization and serves eligible centers throughout their region. All pre‐service and in‐service consultant training, supervision, model fidelity checks, and so forth are centralized though the university contract holder to maintain model fidelity and quality. The IECMHC program leaders are licensed psychologists, and each have over 15 years of experience in training and supervising consultants and in administering an IECMHC program. Please see Heller et al., 2011 for a full explanation of the development of this IECMHC model.

All early education sites that serve publicly funded children are eligible for services. Sites may request IECMHC through an online portal or by calling a centralized number. Requests are received by the primary university contractor staff who connects the requestor with the consultants in the appropriate region. Early education sites may also be contacted by a consultant to determine if they are interested in services. Sites that are rated lower on the statewide accountability system, sites that have higher numbers of children receiving public funding (i.e., child care assistance, foster care), and sites that are new to consultation are given priority if a waitlist exists.

### 1.4 Program theory of change

The TIKES program follows the IECMHC theory of change from the Center of Excellence for Infant and Early Childhood Mental Health Consultation (Georgetown University Center for Child and Human Development, n.d.) that posits the direct effects of an IECMHC program are realized by increasing consultees’ capacity to promote infant and early childhood mental health, with indirect effects potentially including specific early care and education program adjustments to better meet the needs of young children and their families, reductions in disparities, and various specific child and family positive impacts. Moreover, the consultees’ and consultants’ characteristics and backgrounds (e.g., education, race, experience) likely impact how they engage in consultation (e.g., relationship development, readiness for consultation), and this engagement impacts direct (e.g., consultee's ability to support IECMH) and indirect (e.g., changes in classroom or program functioning, reduction in child mental health problems, reduction in child expulsion, etc.) outcomes.

Prior research on this IECMHC program demonstrated that after six months of consultation, teacher‐child interactions improved in the Emotional Support and the Classroom Organization domains of the CLASS‐PK (the Instructional Support domain was not measured) and teachers reported higher teaching self‐efficacy and higher competence in supporting children's social emotional development (Heller et al., 2011, 2012). These outcomes are all components of the direct effects of the theory of change, as they are associated with improved ability to support infant and early childhood mental health. Teachers’ CLASS scores, teaching self‐efficacy, and competence remained elevated six months after consultation had ended (Heller et al., 2012) further supporting the impact of this IECMHC model. Additionally, teachers rated their relationships with their MHC positively, demonstrating that this program's MHCs have been successful in forging strong positive relationships with staff (Heller et al., 2011), which is thought to impact engagement in the IECMHC program.

### 1.5 Study hypotheses

The negative outcomes associated with exclusionary discipline practices (e.g., lack of engagement in school and later involvement in the juvenile justice system) are of great concern (American Psychological Association Zero Tolerance Task Force, 2008; Fabelo et al., 2011; Nicholson‐Crotty et al., 2009); therefore, this study aims to further elucidate mechanisms of action related to potential reductions in expulsion risk after six months of mental health consultation. Specifically, the current study aims to examine potential short term direct impacts for teachers participating in the IECMHC program, which may be associated with reduced expulsion risk for students. The specific study hypotheses were that participating in six months of this IECMHC program will be associated with the following:

- A decrease in teacher reported child behavior difficulties (e.g., child behavior that is difficult for the teacher to manage, child behavior that interferes with classroom functioning, child behavior that interferes with children's learning, and child behavior that puts a burden on the teacher or the class as a whole).
- A decrease in teacher reported child social emotional concerns (e.g., emotion regulation difficulties, inattentiveness).
- An increase in teacher reported child resilience behaviors (e.g., ability to self‐regulate, positive attachment relationships).
- A decrease in level of teacher reported child risk for expulsion (e.g., teacher hopelessness, teaching stress, and teacher fear of accountability as related to child behavior).

Additionally, we were interested in examining whether we might be able to identify teachers who were more or less likely to see benefits from participation in mental health consultation services. Prior research in early childhood education settings has indicated that higher levels of general teacher stress is associated with lower quality teacher‐child interactions (Hamre & Pianta, 2004) and poorer classroom management (Li‐Grining et al., 2010). Additionally, research has suggested that the level of teacher stress (i.e., stress related to factors outside of the teacher child relationship) prior to receiving professional development is associated with lower intervention uptake and may index a readiness to engage in intervention (Li‐Grining et al., 2010; Sandilos et al., 2018). However, teaching stress (i.e., stress related to a specific child) has not been examined in the context of an IECMHC intervention. Given that, we examined whether child‐specific teaching stress assessed pre‐consultation moderated changes in teacher‐perceived child‐level or classroom‐level outcomes from pre‐ to post‐consultation.

## 2 METHODS

The data used in the current study were collected across Louisiana where the TIKES IECMHC program is implemented as part of ongoing program evaluation, and data were collected by the mental health consultants as part of the consultation intake process. The consultants administered the measures at the start and again at the end of the six‐month consultation period. The consultants asked teachers to complete one measure for the entire class, the classroom Strengths and Difficulties Questionnaire Impact Supplement (SDQ‐IS). Based on the responses to that questionnaire, the teacher completed three additional questionnaires on the three children rated as the most difficult in the classroom (hereafter referred to as index children). These were the individual SDQ, the Deveraux Early Childhood Assessment (DECA), and the Preschool Expulsion Risk Measure (PERM). This same process was repeated at the end of the consultation; however, the teachers reported on the same three children that they had completed questionnaires on in the pre‐assessment regardless of the children's ratings on the post‐assessment classroom SDQ‐IS. Participation in the program evaluation data collection was voluntary at both the center and teacher level and did not impact the center's or teacher's ability to receive consultation services. Data were collected as a component of the program's ongoing evaluation, which precluded the use of a control group. However, as consultants initiated and completed IECMHC services at all time points throughout the year, it is unlikely that differences in teacher perceptions would be due to children settling into classroom and relationships being formed between teachers and children across the course of a typical “school” year. This study was approved by the Tulane University Institutional Review Board.

### 2.1 Data collection

Beginning in 2017 and continuing through February of 2020, program consultants invited any childcare center starting a six‐month round of consultation to participate in this evaluation. Participation was completely voluntary and centers or teachers who did not wish to participate still received consultation. All centers that requested consultation or that were recruited to participate in consultation during this period were asked to participate in the evaluation. Across 198 centers served during this period, only four declined to participate in the evaluation. Data were not collected on whether teachers randomized to participate declined participation. Consultants collected pre‐ and post‐evaluation data at 194 centers, from 415 teachers in 415 randomly chosen classrooms, and on 1241 index children. Two classrooms were randomly selected from each center with seven or fewer classrooms, and three classrooms were randomly selected from each center with eight or more classrooms. Center directors and each individual teacher could decline participation in data collection and still receive IECMHC services with no penalty.

Pre‐assessment questionnaires were administered between the first and third MHC visit and post‐assessment questionnaires were administered between the 10th and 12th (final) MHC visit. The MHC administered the measures at the pre‐ and post‐assessment.

### 2.2 Participants

Data were available for between 670 and 1241 children (18 months to approximately 4 years old) who attended 194 centers. Centers were predominantly rated as Approaching Proficient (23.2%) or Proficient (28.9%) on a statewide rating scale that includes Unsatisfactory, Approaching Proficient, Proficient, and Excellent as categories. Centers had between one to four (39.2%), five to seven (38.1%), eight to eleven (18.6%), and 12 or more (4.1%) classrooms total. Children were in a total of 415 classrooms, with an average of 11 children in each classroom at pre‐ and post‐consultation. One teacher reported per classroom (teacher *N* = 415); no demographic or descriptive data were collected from teachers directly. Children were primarily in two (28.8%) or 3‐year‐old (30.7%) classrooms. Additionally, most children were with the same teacher (61.0%) or in the same classroom (78.3%) pre‐ and post‐consultation. The children identified as an index child at the pre‐assessment remained an index child at the post‐assessment even if they were no longer rated as one of the most difficult children in the classroom.

In some cases, post‐assessment data could not be collected. A primary reason for missing post‐assessment data was due to the COVID‐19 pandemic during which many centers suspended services, resulting in the inability to collect post‐assessment data (11.6%). The other primary reasons for missing post‐assessment data were due to a parent decision to remove the child from a center not at the request of the center (i.e., not due to expulsion or suspension), including the family moving (8.5%) or the child leaving the center to attend a school‐based early education program or Head Start (6.9%). While we recognize the importance of child demographic data and its association with potential expulsion risk, demographic data such as sex, race, and ethnicity were not collected on the index children for this project.

### 2.3 Measures

#### 2.3.1 Child behavior difficulties

The extent and impact of child behavior problems in the classroom was assessed with the Strengths and Difficulties Questionnaire‐Impact Supplement (SDQ‐IS; Goodman, 1999). For each child on their roster, teachers indicated whether the child had difficulties in one or more of four areas (i.e., emotions, concentration, behavior, or being able to get along with other people). A classroom‐level behavioral problems score was calculated by summing the teacher's rating of the difficulty level of each child in the classroom on a 4‐point Likert scale, ranging from not difficult to severe difficulties. For the three children with the highest scores (i.e., the index children) on the classroom‐level behavioral problems score, the teacher completed an additional five SDQ‐IS questions. These five questions inquire in more detail about the impact of the behavioral difficulties on the child and class (e.g., Does the behavior interfere with classroom functioning? Does the behavior interfere with child's learning? Does the behavior put a burden on you or the class as a whole?). Predictive validity for the SDQ has been demonstrated (Goodman & Scott, 1999).

The Impact Supplement score has been demonstrated to better discriminate between clinical and community samples than the total SDQ difficulties score (which is a 25‐item behavioral screening questionnaire; Goodman, 1997). The Impact Score has also been demonstrated to be a better predictor of service use and request for additional help when the informant was a parent or primary caregiver (Goodman, 1999; Janssens & Deboutte, 2009).

#### 2.3.2 Child resilience and protective factors

Children's resilience and protective factors were assessed using teacher reports on the Devereaux Early Childhood Assessment (DECA). The 36‐item DECA Toddler (DECA‐T; Powell et al., 2007) is appropriate for use with children 18–36 months. The 38‐item DECA Preschool‐Second Edition (DECA‐P2; LeBuffe & Naglieri, 2012) is used with children who are three to five years old. The DECA asks teachers to report the frequency with which a child exhibits certain resilient and challenging behaviors. Teachers respond on a five‐point Likert scale ranging from never to very frequently. Both the Toddler and Preschool versions of the DECA consist of three subscales: Attachment/Relationships, Initiative, Self‐Regulation—as well as a Total Protective Factors scale, onto which all three subscales load. Teachers completed a DECA for each of the three index children in their classrooms. DECA scores are standardized T scores, with a mean of 50. Scores from 41 to 59 are considered “Typical” and scores of 60 and above are considered “Strengths.” For initiative, Attachment, and Self‐Regulation as well as the umbrella Protective Factors scale, higher scores indicate greater competency in those areas. For Behavioral Concerns, which is not standardized, higher scores indicate more problem behaviors. The DECA is based on resilience research and is a nationally standardized, reliable, and valid measure (Barbu et al., 2013).

#### 2.3.3 Social emotional concerns

Preschool‐aged children's social emotional concerns were also assessed on the DECA. In addition to resilience and protective factor scales, the Preschool DECA contains an additional scale which examines Behavior Concerns. Teachers reported on social‐emotional concerns on a 5‐point Likert scale assessing frequency of concerning behaviors, ranging from never to very frequently. For Behavioral Concerns, which is not standardized, higher scores indicate more problem behaviors.

#### 2.3.4 Risk for expulsion

Children's risk for expulsion, as well as teacher stress due to children's behavior, was assessed using teacher report on the Preschool Expulsion Risk Measure (PERM; Gilliam & Reyes, 2018). The PERM consists of twelve statements about child behavior that the teacher is asked to rate on a 5‐point Likert scale, ranging from strongly disagree to strongly agree. The teacher completed a PERM for each of the three index children. Factor analysis confirmed the PERM's four factor structure—Classroom Disruption (how much the child's behavior interferes with the class), Fear of Accountability (concern about being held responsible for the child's behavior or outcomes of child's behavior), Hopelessness (sense that the child's behavior is unlikely to improve), and Teaching Stress (teacher's stress due to the child's behavior). (N.B.: While the PERM actual scale name is “teacher stress” we are using it to measure “teaching stress” as defined in the introduction, so for readability we will use the term “teaching stress” to denote this scale throughout the paper). Research (Gilliam & Reyes, 2018) has demonstrated good internal consistency and test‐retest reliability as well as concurrent and discriminant validity for the PERM, establishing that the PERM is an appropriate tool for understanding and addressing expulsion risk in preschool settings.

### 2.4 Data analytic plan

Multilevel linear modeling (MLM) was used to evaluate the differences in child and classroom level outcomes across pre‐ and post‐consultation. MLM was completed with the mixed procedure in IBM SPSS Statistics for Windows, Version 27 (IBM Corp, Armonk, NY). The default estimation method, restricted maximum likelihood estimation, was used. The dependent variables were child and classroom level outcomes. Time was coded as a dichotomous independent variable (pre = 0, post = 1). A three‐level model was used for child‐level outcomes, where children's scores were nested within individuals (*N* = 1241), individuals were nested within classrooms (*N* = 415), and classrooms were nested within centers (*N* = 194). Specifically, we used the equations below:

$$Y_{ijk\;}=\beta_{0}+\beta_{1}TIME_{i}+X_{jk}+\mu_{jk}+e_{ijk}.$$

Here, Y reflects the score (e.g., DECA, PERM) of individual child *i* in class *j* in center *k*. TIME reflects the individual‐level dichotomous pre/post time variable, and X reflects classroom‐level controls (age of classroom and number of children in classroom). *μ* denotes the random intercepts for classroom and center, and *e* denotes the error term. The coefficient of interest is *β_1,_* which reflects whether scores on Y were significantly different between pre and post, taking into account classroom and center effects.

For classroom‐level behavior problems, a two‐level model was used, with classroom level *j* behavior problems nested within center *k*:

$$Y_{jk\;}=\beta_{0}+\beta_{1}TIME_{jk}+X_{jk}+\mu_{k}+e_{jk}$$

Here, the coefficient of interest is again *β_1,_* which reflects whether classroom‐level behavior problems of classroom *j* in center *k* were significantly different between pre and post, considering covariates and center effects.

Finally, to evaluate potential moderating effects of Time 1 teaching stress (i.e., stress due to the child's behavior) on change in scores from Time 1 to Time 2, interactions between pre‐consultation teaching stress and time were modeled, along with main effects. For these analyses, we used the following equation:

$$\begin{array}{ccc} Y_{ijk\;}= & & \beta_{0}+\beta_{1}TIME_{ijk}+\beta_{2}STRESS_{ijk}\\ & & +\beta_{3}TIMExSTRESSi_{ijk}+X_{jk}+\mu_{jk}+e_{ijk}. \end{array}$$

In this model, the score Y of individual child *i* in classroom *j* in center *k* was modeled as predicted by time (pre/post), pre‐consultation teaching stress (Grand Mean centered), and a time‐by‐stress interaction, along with X—classroom‐level covariates of age of classroom and number of students in classroom; *μ—*random intercepts for both classroom *j* and center *k*; and error terms *e*. In these models, the coefficient of interest was *β_3_* which, if significant, would suggest that children's change over time varied by teacher's pre‐consultation stress. All significant interactions were decomposed to interpret effects.

## 3 RESULTS

### 3.1 Descriptive analysis

Table 1 summarizes the descriptive data for child, class, and center‐level characteristics, as well as pre‐ and post‐consultation means for variables of interest. Distribution of outcome measures was consistent with normality assumptions (skew and kurtosis values < 3.0).

**TABLE 1:** Descriptives of center, classroom, and child characteristics and outcomes pre‐ and post‐consultation.

	Pre consultation			Post consultation		
	*n*	M (SD) or %	Pooled M^*^	*n*	M (SD) or %	Pooled M^*^
**Center, classroom, and child characteristics**						
Proficiency rating of center	106					
Unsatisfactory	4					
Approaching Proficient	45					
Proficient	56					
Excellent	1					
Number of classrooms in center	194					
1–4 classrooms	76	39.2%				
5–7 classrooms	74	38.1%				
8–11 classrooms	36	18.6%				
12 or more classrooms	8	4.1%				
Age range of classrooms per child	1241					
Toddler (18 months and older)	180	14.5%				
Two‐year‐olds (24 months and older)	358	28.8%				
Three‐year‐olds (36 months and older)	381	30.7%				
Four‐year‐olds (48 months and older)	143	11.5%				
Mixed age group	179	14.4%				
Child was with the same teacher				450	61.0%	
Child was in the same classroom				578	78.3%	
Number of children in each classroom	413	10.73 (4.09)		311	10.48 (3.81)	
**Child level outcomes**						
SDQ‐IS Total Behavior Problems, Sum Score	1241	7.27 (3.85)	7.26	736	6.10 (3.85)	6.21
DECA Initiative, T‐Score	1236	44.78 (10.80)	44.78	736	48.81 (10.68)	48.25
DECA Attachment, T‐Score	1236	44.98 (10.96)	44.99	736	48.98 (10.54)	48.45
DECA Self Control, T‐Score	1236	40.92 (10.60)	40.92	736	44.84 (10.81)	44.26
DECA Total Protective Factors, T‐Score	1236	42.71 (10.43)	43.72	736	47.19 (10.73)	46.56
DECA Total Behavioral Concerns, T‐Score	670	60.05 (8.44)	58.94	443	56.19 (9.39)	55.99
PERM Classroom Disruption, Mean Score	1241	3.11 (1.39)	3.11	737	2.57 (1.36)	2.64
PERM Fear of Accountability, Mean Score	1241	2.30 (1.23)	2.30	737	1.95 (1.13)	2.00
PERM Hopelessness, Mean Score	1241	2.13 (1.13)	2.13	737	1.91 (1.06)	1.93
PERM Teacher Stress, Mean Score	1241	2.29 (1.39)	2.29	737	1.94 (1.24)	2.02
**Classroom level outcomes**						
SDQ‐IS Total Behavior Problems, Sum Score	412	9.35 (5.27)	9.36	306	7.61 (5.04)	7.66

* Pooled M reports estimate from 20 imputations, n = 1241.

Interclass correlation coefficients (ICCs) were calculated for each of the variables at classroom and center level (see Table 2). For child level outcomes, ICCs ranged from .02 to  .11 at the center level and  .21 to  .34 at the classroom level, suggesting 2%–11% of the variance in outcomes was due to variation between centers, and 21%–34% of the variance in outcomes was due to variation between classrooms. For the classroom level outcome of SDQ‐IS problem behaviors, the ICC was .41 at the center level, suggesting 41% of the variance in classroom‐level problem behaviors was attributable to variation between centers.

**TABLE 2:** Interclass correlations of child and classroom level outcomes.

	Center	Classroom
	ICC	ICC
*Child level outcomes*		
SDQ‐IS Total Behavior Problems, Sum Score	.02	.34
DECA Initiative, T‐Score	.03	.24
DECA Attachment, T‐Score	.02	.27
DECA Self Control, T‐Score	.06	.28
DECA Total Protective Factors, T‐Score	.05	.28
DECA Total Behavioral Concerns, T‐Score	.10	.21
PERM Classroom Disruption, Mean Score	.07	.29
PERM Fear of Accountability, Mean Score	.05	.28
PERM Hopelessness, Mean Score	.11	.23
PERM Teacher Stress, Mean Score	.06	.32
*Classroom level outcomes*		
SDQ‐IS Total Behavior Problems, Sum Score	.41	

Child level pre‐ and post‐consultation data for all outcomes excepting DECA Behavioral Concerns were missing at random (Little's MCAR test: *χ* ^2^ = 88.98, *p* = .36): there were 41% of total cases missing post‐consultation SDQ‐IS child behavior problems, DECA self‐control, DECA attachment, DECA self‐regulation, DECA protective factors, PERM hopelessness, PERM fear of accountability, PERM classroom disruption, and PERM teacher stress. DECA behavioral concerns were not expected to be missing at random because they are only calculated for children ≥ 36 months; 46% of children were missing these data pre‐consultation and 64.3% post‐consultation. Missing variable analysis of SDQ‐IS classroom‐level behavior problems indicated cases were missing at random (Little's MCAR test: χ2 = 13.18, *p* = .11), with 25% of classrooms missing post‐consultation SDQ‐IS behavior problems. Given the predominant pattern of missing at random, we used multiple imputation (MI; 20 imputations) to generate pooled estimates, which are the values reported in results; all pre‐ and post‐consultation data were included in imputation. Of note, no major discrepancies in patterns of pre‐post main effect results were observed across the pooled multiple imputation results versus listwise deletion. Specific patterns of moderation by teaching stress, discussed below, varied slightly across MI versus listwise deletion, though the broad patterns—teaching stress differentially predicting change over time—remained consistent across methods of handling missing data. Table 3 presents bivariate correlations between child‐level variables of interest at the pre‐consultation time point.

**TABLE 3:** Bivariate correlations between child variables of interest at Time 1.

	Child SDQ	DECA initiative	DECA attach	DECA self‐regulation	DECA total protect	DECA behavior concerns	PERM class disrupt	PERM account‐ability	PERM Hopeless
DECA Initiative	−.32^***^								
DECA Attachment	−.27^***^	.70^***^							
DECA Self‐Regulation	−.52^***^	.59^***^	.51^***^						
DECA Total protective	−.43^***^	.88^***^	.86^***^	.81^***^					
DECA Behavioral concerns	.61^***^	−.43^***^	−.35^***^	−.68^***^	−.58^***^				
PERM Class disrupt	.67^***^	−.23^***^	−.22^***^	−.52^***^	−.37^***^	.60^***^			
PERM Accountability	.54^***^	−.18^***^	−.18^***^	−.43^***^	−.30^***^	.54^***^	.66^***^		
PERM Hopeless	.48^***^	−.30^***^	−.29^***^	−.38^***^	−.38^***^	.45^***^	.49^***^	.50^***^	
PERM Teacher stress	.55^***^	−.22^***^	−.22^***^	−.44^***^	−.34^***^	.51^***^	.68^***^	.60^***^	.59^***^

*Note*: ^***^ < .001; pooled results from twenty imputations reported; *n *= 1241.

### 3.2 Differences from pre‐ to post‐consultation

Regarding main effects of time, covarying classroom size and age of classroom, all outcomes were significantly different from pre‐ to post‐consultation (see Table 4), with behaviors and protective factors changing in the directions predicted in hypotheses. At post‐consultation, teachers reported significantly higher child resilience and protective factors (i.e., total protective factors as well as the subscales of self‐regulation, attachment, and initiative), lower child‐ and classroom‐level behavioral concerns (i.e., DECA total behavior concerns, SDQ‐IS child and classroom behavior problems), and lower child‐level factors related to an increased likelihood of expulsion (i.e., PERM scales of classroom disruption, teacher stress, teacher hopelessness, and teacher fear of accountability, all scores related to the child's behavior). Thus, all hypotheses were supported in our analyses. Class size also was consistently associated with poorer outcomes, with larger classes associated with higher problem behaviors and lower protective factors across all outcomes of interest. Age of classroom was not associated with child outcomes.

**TABLE 4:** Results of multilevel regression predicting change in child and classroom level outcomes from pre‐ to post‐consultation.

	Estimated marginal means						
Fixed effects	Pre	Post	Estimate	SE	*t*	*p*‐value	95% CI
*Child level*							
SDQ‐IS Total Behavior Problems, Sum Score	7.31	6.32	.99	.15	6.63	.000	.70, 1.28
DECA Initiative, T‐Score	44.79	48.13	−3.34	.44	07.64	.000	−4.20, −2.48
DECA Attachment, T‐Score	44.90	48.30	−3.40	.44	−7.76	.000	−4.26, −2.54
DECA Self‐Regulation, T‐Score	40.78	43.99	−3.20	.40	−7.91	.000	−3.99, −2.41
DECA Total Protective Factors, T‐Score	42.62	46.32	−3.70	.40	−9.21	.000	−4.49, −2.91
DECA Total Behavioral Concerns, T‐Score	59.00	56.22	2.78	.38	7.32	.000	2.04, 3.53
PERM Classroom Disruption, Mean Score	3.12	2.68	.44	.05	8.22	.000	.34, .54
PERM Fear of Accountability, Mean Score	2.32	2.03	.30	.05	6.20	.000	.20, .39
PERM Hopelessness, Mean Score	2.12	1.92	.20	.04	4.58	.000	.14, .28
PERM Teacher Stress, Mean Score	2.28	1.99	.29	.05	5.69	.000	.19, .39
*Classroom level*							
SDQ‐IS Total Behavior Problems, Sum Score	9.54	7.83	1.71	.18	9.63	.000	1.36, 2.06

*Note*: Reporting pooled estimates from 20 imputations. Coefficient estimates are unstandardized. Time was coded as 0 = pre, 1 = post. Analyses were covaried by age of classrooms and number of children in each classroom. DECA scores are standardized T scores, with a mean of 50; scores from 41 to 59 are considered “Typical” and 60 and above is considered “Strength.” Initiative, Attachment, and Self‐Control are Protective Factors, with higher scores indicating more competencies. For Behavioral Concerns, higher scores indicate more problem behaviors. The SDQ‐IS and PERM are not standardized; higher scores indicate more problem behaviors or stress.

Regarding main effects of pre‐consultation teaching stress related to the child's behavior, covarying classroom size and age of classroom, all outcomes were significantly associated with pre‐consultation teaching stress (*p* < .001), with higher teaching stress pre‐consultation associated with poorer outcomes post‐consultation.

### 3.3 Differences from pre‐ to post‐consultation conditional on pre‐consultation teaching stress

Given patterns of main effects, we were interested in whether changes in child‐ and classroom‐level outcomes from pre‐ to post‐consultation varied by teachers’ reports of child related teaching stress as measured by the PERM subscale, Teacher Stress, at the initiation of the consultation. We modeled pre‐consultation teaching stress by time interactions across all outcomes, again covarying classroom size and age of classroom, along with main effects.

**FIGURE 1:**
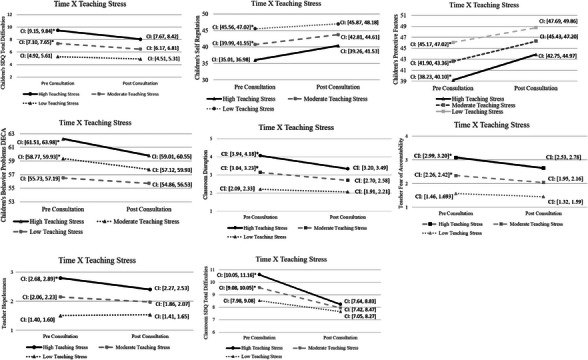
Teaching stress moderates improvements in behavior problems and protective factors pre‐post consultation. *Note*: Means and confidence intervals for outcomes at pre‐ and post‐ consultation that demonstrated significant interactions of time by teaching stress (see Table 5) are graphed at the mean and ±1 SD of pre‐consultation teaching stress. CI: confidence intervals; *indicates non‐overlapping confidence intervals pre‐post consultation.

Significant teaching stress by time interactions were observed for child behavior problems on the SDQ and DECA behavior problems; resilience and protective factors of the DECA self‐regulation subscale and the overall DECA protective factors scale; and risk for expulsion as measured by teacher reported fear of accountability, hopelessness, and classroom disruption on the PERM (see Table 5, Figure 1). Teaching stress by time interactions were also observed for classroom‐wide behavior problems. Significant interactions suggest that change over time in each of the above outcomes varied by teaching stress level at the start of the consultation period. Only main effects of time and teaching stress were observed for the protective factors subscales of child attachment and initiative as measured on the DECA, with no significant time by teaching stress interactions observed for these specific resilience and protective factors outcomes (*p*s > .09). Main effects of time and pre‐consultation teaching stress with non‐significant interactions by teaching stress for these outcomes suggest that change over time was observed, and that higher teaching stress at intake was associated with poorer outcomes, but that change over time did not vary by teaching stress.

**TABLE 5:** Fixed effects from multilevel models demonstrating pre‐teaching Stress × Time interactions for outcomes of interest.

Predictor	Child SDQ behavior problems (Est (SE))	DECA self‐regulation	DECA protective factors (Est (SE))	DECA behavior problems (Est (SE))	PERM fear of accountability (Est (SE))	PERM hopelessness (Est (SE))	PERM classroom disruption (Est (SE))	Classroom difficulties SDQ (Est (SE))
Intercept	5.76 (.48)^***^	43.93 (1.32)^***^	47.63 (1.26)^***^	57.30 (.92)^***^	1.98 (.15)^***^	1.78 (.14)^***^	2.55 (.16)^***^	3.48 (.62)^***^
**Time: Pre**	.89 (.15)^***^	−2.94 (.41)^***^	−3.68 (.41) ^***^	1.61 (.32)^***^	.29 (.05)^***^	.18 (.05)^***^	.43 (.06)^***^	1.62 (.20)^***^
Time: Post	–	–	–	–	–	–	–	–
**Age (Toddler)**	−.98 (.46)^*^	.10 (1.24)	.611 (1.65)	.46 (.87)	−.10 (.14)	−.18 (.13)	−.30 (.15)^*^	−1.51 (.52)^**^
Age = 2 years	−.63 (.40)	1.27 (1.08)	1.00 (1.02)	−.41 (.87)	−.12 (.12)	−.09 (.11)	−.21 (.13)	−1.48 (.45)^**^
Age = 3 years	−.43 (.38)	.41 (1.07)	1.36 (1.01)	−.71 (.74)	−.23 (.12)	−.16 (.11)	−.14 (.12)	−2.05 (.46)^***^
Age = 4 years	.82 (.48)	−.22 (1.31)	1.27 (1.26)	.39 (.91)	.11 (.15)	.02 (.13)	.10 (.16)	−1.69 (.54)^**^
Age = Mixed	–	–	–	–	–	–		–
**T1 Stress**	1.14 (.09)^***^	−2.39 (.26)^***^	−1.77 (.24)^***^	1.47 (.18)^***^	.43 (.03)^***^	.32 (.03)^***^	.46 (.03)^***^	.21 (.11)
**# Children**	.09 (.03)^**^	−.05 (.09)	−.20 (.08)^*^	.05 (.06)	.01 (.01)	.03 (.01)^**^	.02 (.01)^*^	.54 (.04)^***^
**T1 Stress × Time**	.38 (.10)^***^	−1.05 (.29)^***^	−.72 (.27)^**^	.613 (.21)^**^	.11 (.03)^***^	.15 (.03)^***^	.20 (.04)^***^	.54 (.13)^***^

*Note*: T1 Stress: Pre‐consultation teaching stress reported on Preschool Expulsion Risk Measure, which was Grand Mean centered; # children: covariate of size of class; ^*^ *p <* .05, ^**^ *p <* .01, ^***^ *p <* .001.

To decompose significant interactions, we estimated means of variables of interest at pre‐ and post‐consultation at the mean (0) and ±1 SD of teaching stress at T1, corresponding to low and high teaching stress. See Figure 1 for plots of all significant interactions. We interpreted non‐overlapping confidence intervals from pre‐ to post‐consultation as evidence of significant change from pre‐post.

Children's total difficulties as reported by teachers on the SDQ changed significantly pre‐ to post‐consultation among children of teachers who reported high and moderate levels of teaching stress at the initiation of treatment. Similarly, for child self‐regulation on the DECA, significant pre‐ to post‐changes were observed for children whose teachers were experiencing high and moderate teaching stress. For protective factors, changes were significant for all children, as suggested by non‐overlapping confidence intervals pre‐post; however, as evidenced by plotted means and confidence intervals, it is notable that the average child of teachers who reported the highest levels of teaching stress pre‐consultation moved from an area of concern regarding protective factors (T score < 41) to overlapping with the typical category (T score = 41–59) post‐consultation. For DECA behavior problems, significant differences pre‐post again were observed only among children whose teachers reported high and moderate teaching stress at initiation. As with protective factors, for children in classrooms with teachers with high levels of teaching stress, scores moved from above the clinical cut‐off pre‐consultation (T score > 60) to below this cut‐off at the post‐consultation time point, suggesting clinically meaningful change.

Regarding risk for expulsion assessed with the PERM, significant improvements in classroom disruption and fear of accountability were observed among teachers reporting high and moderate levels of teaching stress at initiation of the consultation; teacher hopelessness improved significantly only among teachers reporting high teaching stress at pre‐consultation.

For classroom‐level behavior problems assessed with SDQ scores, change from pre‐post was observed across both high and moderate levels of teaching stress. Critically, at the post‐consultation time point, classroom‐level SDQ scores were not significantly different from one another across teachers who reported high, moderate, and low levels of stress at initiation, as evidenced by overlapping confidence intervals at post‐consultation

Taken together, these patterns suggest that significant change over time in child‐level behavior problems and protective factors, and teacher factors related to risk for expulsion such as hopelessness, fear of accountability, and classroom disruption, were more pronounced among teachers reporting higher levels of teaching stress prior to initiation of consultation. Additionally, corresponding to main effects of teaching stress, results suggest that teachers who reported low stress about the child's behaviors pre‐consultation consistently reported lower levels of poor outcomes across time, compared to teachers reporting moderate to high levels of teaching stress. Classroom‐level behavior problems assessed with the SDQ also changed most significantly for teachers reporting the highest levels of teaching stress pre‐consultation; there were no significant differences across teaching stress levels at post‐consultation in classroom‐level behavior problems.

## 4 DISCUSSION

All four research objectives were supported. The analyses demonstrated a significant: (1) decrease in teacher reported child behavior difficulties (e.g., child behavior that interferes with the classroom, child behavior that interferes with children's learning); (2) decrease in teacher‐reported child social emotional concerns (e.g., emotional regulation difficulties, inattentiveness); (3) increase in teacher reported child resilience and protective factors (e.g., ability to self‐regulate, positive attachment relationships); and (4) decrease in level of teacher reported child risk for expulsion (e.g., teacher hopelessness, teaching stress, and teacher fear of accountability as related to child behavior) after receiving six months of mental health consultation services. It is noteworthy, although not surprising, that teachers in larger classrooms reported higher rates of challenging behaviors and lower rates of protective behaviors.

When we examined our findings based on teaching stress (i.e., stress proximal to a specific child), teachers reporting high teaching stress reported decreases in challenging behavior and increases in protective behavior post consultation, and for protective factors and challenging behavior alike, the means moved from the clinical to the typical range. Additionally, teachers reporting high teaching stress demonstrated a significant change in how they reported classroom level difficulty after consultation. Furthermore, post‐consultation classroom level SDQ scores were similar across all teaching stress levels suggesting that the intervention “leveled the playing field’ for classroom‐level behavior problems, regardless of initial levels of teaching stress.

Prior research has demonstrated that highly stressed teachers are associated with less positive student relationships (Ansari et al., 2022) and more disruptive student behaviors (Herman et al., 2017). Moreover, teachers who have requested expulsion within the past year more explicitly linked their stress to the behavior of an individual child, whereas teachers who had not recently requested expulsion reported sources of stress that were not specific to an individual child (Zinsser et al., 2019). Encouragingly, our findings suggest that teachers with high teaching stress, and the children in their care, benefited most from consultation as evidenced by greater reductions in expulsion‐related factors, more improvements in behavior problems, and stronger gains in protective factors. However, their overall scores remained lower than those of teachers reporting less teaching stress. The impact of child specific teaching stress on IECMHC outcomes provides additional information about how mental health consultation works, how it may be associated with lowered expulsion risk, and has implications for the strategies and areas of focus in MHC. Through interaction with the MHC, the teachers may have been able to implement more effective classroom behavior management practices, follow behavior plans developed with the MHC, and access other supports that could potentially decrease challenging behaviors in the classroom while also fostering children's resilience and protective factors. This lends credence to the programmatic nature of the evaluated model of IECMHC and its benefits for the individual teachers and children participating in the program. Additionally, it highlights the importance of identifying teachers with higher levels of teaching stress who may be in greater need of IECMHC interventional supports.

### 4.1 Limitations

While causal inferences linking IECMHC to changing teacher perceptions cannot be made based on these findings, correlational patterns are present that support this six‐month model of IECMHC's expected outcomes in teachers’ perceptions of student protective factors and behavior difficulties in the predicted direction. Other limitations in the current study include a lack of demographic factors that have been linked to early childhood expulsion (e.g., sex, race, and ethnicity). As demographic data were not collected, any impact that this IECMHC program had on reducing race‐ and sex‐related disparities in early childhood expulsion or how these factors may be associated with teaching stress, implicit bias, and evaluation outcomes could not be explored. Teacher‐specific data, such as years of experience, age, and education level, were not collected either. These factors may also influence teachers’ stress and their perceptions of children's behavior. Additionally, as these data were collected as part of overall program evaluation, there is not a control group to which outcomes could be compared. As many early childhood programs follow the schedule of a school system, moving children up to new classrooms in the fall, if data were collected only at the beginning of the academic year (e.g., August, September), then it may be expected that child and class behavior difficulties would decrease after six months as children developed relationships with teachers and peers and learned new routines and expectations. However, this IECMHC model operates year‐round, and consultants begin and end six‐month consultation periods in every month of the year depending upon their unique schedules. This makes it unlikely that the changes found were due to children settling into a new routine after starting in a new classroom with a new teacher. While this study examined teaching stress (stress related to a specific child), it did not measure the many stressors impacting ECE teachers including low compensation, lack of benefits and professional respect as well as the multiple physical, cognitive, and emotional demands of the role (i.e., general teacher stress). Future studies should consider the impact of teacher stress and teaching stress within the same sample, including how external stressors that a teacher experiences (i.e., teacher stress) may directly impact a teacher's relationship with and perception of the children in his or her care. Teacher stressors not related to a specific child may contribute to the teaching stress (stress related to a specific child) that a teacher experiences. A final limitation is that all data were teacher reported; direct observation of child‐level or classroom‐level behaviors were not collected.

## 5 CONCLUSION

Early childhood education teachers are vital to providing quality care for young children and their ability to provide responsive and warm interactions is necessary to create classroom environments that support children's healthy social emotional development (de Kruif et al., 2000), contributing to long term positive outcomes, fewer behavior difficulties in school, and higher graduation rates (Schweinhart & Weikart, 1981; Raver, 2003). When teachers experience high levels of stress, their ability to provide consistent emotional support, create a positive classroom climate, and use positive behavior management techniques may be hampered (Hamre, 2014; Zinsser et al., 2013). These findings support the hypothesis that IECMHC may reduce expulsion risk by positively influencing teachers’ perceptions of the children in their care. Specifically, after participating in this IECMHC program teachers reported lower levels of perceived child and classroom level behavior difficulties, child social emotional concerns, and less hopelessness, teaching stress, and fear of accountability along with increased perceived child resiliency factors. For IECMHC programs, such as this six‐month model, it may be important for MHCs to address child specific teaching stress and examine how that stress may be impacting teachers’ views on children whose behavior they perceive as challenging. Although teachers with the highest teaching stress demonstrated the largest decrease in perceived child behavior problems and expulsion risk after consultation, they continued to report higher levels of expulsion risk for the identified children when compared to teachers with lower pre‐consultation teaching stress.

To better understand how IECMHC impacts expulsion risk, continued examination of ECE teaching stress along with other ECE teacher characteristics can clarify IECMHC influence on teacher‐perceived child level outcomes. Establishing a causal link between IECMHC programs and changes in teachers’ perceptions of children's behavior and their own stress levels, whether child‐specific stress (i.e., teaching stress) or overall stress, requires a comparison between teachers receiving six months of consultation to those in a control group. IECMHC has been established as a primary method for working with teachers to reduce child expulsion risk (Gilliam, 2005; Gilliam et al., 2016); however, while the present study begins to explore variables that may influence IECMHC, much remains to be learned about the mechanism through which it operates.

## FUNDING INFORMATION

This project was funded via contract by the Louisiana Department of Education, LaGovP.O.# 2000457317.

## CONFLICT OF INTEREST STATEMENT

The authors declare no conflicts of interest.

## Data Availability

The data that support the findings may be available from the corresponding author upon reasonable request if approved by the funder.
